# DNA Base Pair Resolution Measurements Using Resonance Energy Transfer Efficiency in Lanthanide Doped Nanoparticles

**DOI:** 10.1371/journal.pone.0117277

**Published:** 2015-03-06

**Authors:** Aleksandra Delplanque, Dominika Wawrzynczyk, Pawel Jaworski, Katarzyna Matczyszyn, Krzysztof Pawlik, Malcolm Buckle, Marcin Nyk, Claude Nogues, Marek Samoc

**Affiliations:** 1 LBPA, IDA (FR3242), ENS Cachan, 61 Avenue du Président Wilson, F-94235 Cachan, France; 2 Institute of Physical and Theoretical Chemistry, Wroclaw University of Technology, Wybrzeze Wyspianskiego 27, 50–370 Wroclaw, Poland; 3 Institute of Immunology and Experimental Therapy, Polish Academy of Sciences, ul. Weigla 12, 53–114 Wroclaw, Poland; 4 Department of Toxicology, Wrocław Medical University, Borowska 211, 50–556 Wrocław, Poland; CNR, ITALY

## Abstract

Lanthanide-doped nanoparticles are of considerable interest for biodetection and bioimaging techniques thanks to their unique chemical and optical properties. As a sensitive luminescence material, they can be used as (bio) probes in Förster Resonance Energy Transfer (FRET) where trivalent lanthanide ions (La^3+^) act as energy donors. In this paper we present an efficient method to transfer ultrasmall (ca. 8 nm) NaYF_4_ nanoparticles dispersed in organic solvent to an aqueous solution via oxidation of the oleic acid ligand. Nanoparticles were then functionalized with single strand DNA oligomers (ssDNA) by inducing covalent bonds between surface carboxylic groups and a 5’ amine modified-ssDNA. Hybridization with the 5’ fluorophore (Cy5) modified complementary ssDNA strand demonstrated the specificity of binding and allowed the fine control over the distance between Eu^3+^ ions doped nanoparticle and the fluorophore by varying the number of the dsDNA base pairs. First, our results confirmed nonradiative resonance energy transfer and demonstrate the dependence of its efficiency on the distance between the donor (Eu^3+^) and the acceptor (Cy5) with sensitivity at a nanometre scale.

## Introduction

Lanthanide-doped nanoparticles (La-NPs) are of interest in nanoscience because of their unique chemical and optical properties, including large effective Stokes shift, long luminescence lifetimes, narrow emission bands and high photochemical stability [1,2]. Additionally, their low toxicity and high detection sensitivity make them a perfect candidate for biosensing and bioimaging applications [3–5], as well as a promising alternative to well established bioprobes such as organic dyes or quantum dots. Currently, La-NPs are successfully implemented in bioassays [6], drug delivery [7], imaging of cells, tissues and small animals [8], and photodynamic therapy [9]. Since excitation and emission of La-NPs can occur in visible and NIR regions, the problems of biological tissue autofluorescence or phototoxicity can be easily overcome [10]. Although, the NIR excitation through the up-conversion process seems to be more advantageous, the down converted emission has higher quantum yield making the results of spectroscopic measurements less susceptible to the fluctuation in sample concentration or excitation power. One of the main drawbacks, regarding biological applications of La-NPs is their hydrophobicity. Their synthesis usually requires the presence of a hydrophobic organic ligand to stabilize the crystal structure [11]. However, the presence of these ligands on the surface of NPs causes their low solubility in water and unfavorable surface properties. Therefore, an efficient method of transferring La-NPs into aqueous solution is essential to work with biomolecules [12]. A large number of techniques that alleviate the hydrophobic character of La-NPs and simultaneously provide biologically functional moieties, such as carboxyl, hydroxyl, amine or thiol groups have been developed [1]. These techniques can be divided into those making use of ligand oxidation [12], exchange [13–16] or the attraction of molecules with a functional group [17–19] and those that involve the deposition of a functionalized silane layer on the La-NPs surface [20,21]. In the particular case of NaYF_4_ La-NPs synthesized using thermal decomposition reactions [22], oleic acid molecules serve as capping ligands and produce a protective monolayer around the NPs [11,23]. An efficient method to modify surface properties of NaYF_4_-NPs involves selective oxidation of a C-C double bond of oleic acid to produce two carboxylic acids [12,24,25]. Carboxylic groups on the NaYF_4_-NPs surface increase the hydrophilicity of the system and allow biomolecules such as nucleic acids, proteins or antibodies to be immobilized. These may then be conjugated with fluorescent tags and used as luminescence probes for detection of biomolecules and their interactions or bioimaging of cells and tissues [8]. NaYF_4_-NPs doped with various lanthanide ions can be used in Förster/Fluorescence or Luminescence Resonance Energy Transfer (FRET or LRET) as sensitive and selective luminescence probes [26], where trivalent lanthanide ions serve as energy donors [27–31]. The LRET approach usually employed in experiments is an extension of the well-known FRET [32,33] characterized by energy transfer from an excited donor to an acceptor placed in its direct vicinity. However, as a luminescence strong reabsorption process, LRET is independent of the relative distance between donor and acceptor molecules, thus obviating the need for their physical proximity. Thus, to detect intermolecular interactions and to measure distances between fluorescent (bio) molecules [5,6] FRET is more appropriate. The principles of FRET require partial overlapping between the emission spectrum of the donor and absorption spectrum of the acceptor. As FRET is a distance-dependent phenomenon, a short distance between donor and acceptor is crucial and depends on the Förster radius (R_0_—the distance at which 50% of energy is transferred to the acceptor). In the case of lanthanide ions acting as donors, the value of R_0_ can be extended to 7.5 nm because of their spectral properties and thus allows efficient energy transfer in the range of 10–20 nm [27].

We demonstrate that ultra small (ca. 8 nm) NaYF_4_:10%Eu^3+^-NPs can be efficiently transferred into water through oxidation of oleic acid ligands with the Lemieux—van Rudloff reagent (5.7 mM KMnO_4_ and 0.105 M NaIO_4_) [12,24] and its ruthenium III salt analogue (1.57 mM RuCl_3_·x H_2_O and 0.105 M NaIO_4_) [25]. Carboxylic groups on the surface of hydrophilic NaYF_4_:10%Eu^+3^-NPs were functionalized and conjugated with 5’ amine tagged single strand DNA by the formation of covalent bonds at the interface between the La-NPs and the DNA strand. Fluorophore (Cy5) placed at the 5’ extremity of the complementary ssDNA led to the assembly of the energy-acceptor couple for the energy transfer process to take place. Different lengths of DNA (26 bp, 31 bp, 41 bp, 50 bp) were examined in order to optimize the distance for an efficient FRET. Measurements of emission spectra and luminescence lifetime values confirmed the dependence of the FRET efficiency with the donor-acceptor distance; the optimum distance being for the 26bp DNA double strand. In addition, digestion of dsDNA by a restriction enzyme (BamHI) allowed the controlled release of Cy5 from the complex to the solution, leading to the extension of the FRET signal, demonstrating thereby the specificity and functionality of such bio-probes.

## Materials and Methods

### Reagents and Oligonucleotides

All experiments were performed with double distilled water; buffers were filtered before use. N-(3-dimethylaminopropyl)-N′-ethylcarbodiimide hydrochloride (EDC) and N-hydroxysuccinimide (NHS) were purchased from Sigma-Aldrich and GE. Oligonucleotides were purchased from Eurofins Genomics and Sigma.

### Synthesis of NaYF_4_:10%Eu^+3^-NPs and their Transfer to Water

Synthesis of small (ca. 8 nm) NaYF_4_-NPs doped with 10% of Eu^3+^ ions was carried out essentially as described in [11,26]. In order to transfer synthesized NaYF_4_:10%Eu^+3^-NPs into water, we used two protocols: (i) Oxidation with the Lemieux—van Rudloff reagent: a mixture of 2 ml synthesized NaYF_4_:10%Eu^+3^-NPs in cyclohexane, 10 ml cyclohexane, 7 ml *tert*-butanol, 1 ml water and 0.5 ml K_2_CO_3_ aqueous solution (5%) *wt* was placed in a round-bottom flask and stirred at room temperature for 20 min. 2 ml of the freshly prepared Lemieux—van Rudloff reagent (5.7 mM KMnO_4_ and 0.105 M NaIO_4_) was added dropwise. The mixture was stirred at 40°C for 48 hours, centrifuged and then washed with water, acetone and ethanol respectively, then twice with water. NaYF_4_:10%Eu^3+^-NPs were then dispersed in 6 ml water. (ii) Oxidation with RuCl_3_/NaIO_4_: a mixture of 2 ml synthesized NaYF_4_:10%Eu^3+^-NPs in cyclohexane and 21 ml water/ethyl acetate/acetonitrile (3/2/2) was placed in a round-bottom flask and stirred at room temperature. 2 ml of the freshly prepared reagent 1.57 mM RuCl_3_·x H_2_O and 0.105 M NaIO_4_ was added dropwise. The mixture was stirred at room temperature for 2 hours then the aqueous fraction was purified with chloroform several times. The final product was stored at 4°C or used directly for further experiments.

### Bio-conjugation of NaYF_4_:10%Eu^+3^-NPs

NaYF_4_:10%Eu^3+^-NPs previously transferred to water were activated with a mixture of EDC/NHS (ratio 1:1; 0.4 M EDC, 0.1 M NHS) from 30 min to overnight to facilitate the coupling of carboxyl groups to amine-tagged DNA. 8–10 μM ssDNA-NH_2_ was then added to the aqueous solution of NaYF_4_:10%Eu^3+^-NPs and incubated for 2–48 hours. Next, 8–10 μM of the complementary DNA strand in PBS buffer was incubated for additional 2 to 8 hours. Covalent binding of oligonucleotides to the NaYF_4_:10%Eu^3+^-NPs surface was examined with DNA strands labeled with α [^32^P]. Additionally, samples containing dsDNA were digested by the restriction enzyme BamHI for 1–2 hours at 37°C. DNA sequences are listed in S1 Table.

### Sample characterization

Transmission electron microscopy (TEM) images were performed with a FEI Tecnai G^2^ 20 X-TWIN microscope. Emission spectra of samples were recorded with a SPEX FluoroLog-3 (Horiba-Jobin-Yvon) spectrofluorimeter. In order to measure the FRET efficiency we used a home-built system consisting of a commercial tunable Ti:Sapphire laser pumped by a Nd:YAG LOTIS TII laser (Belarus). Luminescence signals, after excitation with 394 nm laser line from second harmonic of Nd:YAG LOTIS TII laser, were detected using a Synapse CCD camera (Horiba-Jobin-Yvon). Fluorescence decay curves, after 394 nm excitation, were recorded for with a photomultiplier (HAMAMATSU R928) output fed into a LeCroy Wave Surfer 425 digital oscilloscope. The decay curves were recorded for the ^5^D_0_→^7^F_2_ emission band in Eu^3+^ ions at 612 nm with wavelength selection performed by a JobinYvon THR1000 monochromator. All the decay curves were fitted with the double exponential model

## Results and Discussion

All experiments were performed with NaYF_4_-NPs doped with 10% Eu^3+^ [11]. Analysis of TEM images showed that the NaYF_4_:10%Eu^3+^-NPs before transfer to water were uniform in size with a very narrow size distribution and had a regular round shape. According to TEM images and data from DLS (Dynamic Light Scattering) (see S1 Fig. and S2 Fig.) their average size was 8 nm. The small size of NPs increases the surface—volume ratio and thus more ions of the doped NPs structure are exposed on their surface [11]. As a consequence doped ions are more easily available for potential interactions with ligands or molecules of interest attached to the surface. This also facilitates the positioning of the ions and their involvement in FRET with bound (bio) molecules. Moreover, the small size of the synthesized NaYF_4_:10%Eu^3+^-NPs improves specificity for binding biomolecules and provides higher control for further applications.

### Transfer to water

Transfer of hydrophobic NaYF_4_:10%Eu^3+^-NPs to water was essential to carry out further experiments with biological molecules. Amongst a range of available and tested methods, we chose transfer into water via oxidation of a hydrophobic ligand that caps NPs [12]. Oleic acid which stabilized the structure of NaYF_4_:10%Eu^+3^-NPs, was oxidized in order to break double C-C bonds into dicarboxylic ligand (azelaic acid). Oxidation was performed either with the Lemieux—van Rudloff reagent (KMnO_4_/NaIO_4_) in a mixture of cyclohexan/*tert-*butanol/water or with the reagent RuCl_3_/NaIO_4_ in a mixture of water/ethyl acetate/acetonitrile [25]. During the reaction changes of color were observed indicating that oxidation had occurred. The reaction with the Lemieux—van Rudloff reagent changed the colour from purple to dark orange. Initial changes were observed after 4–5 hours. Oxidation by the RuCl_3_/NaIO_4_ reagent changed the colour from bright brown at the beginning to dark green at the end of the incubation period (2 hours).

After the purification steps, NaYF_4_:10%Eu^+3^-NPs oxidized with the Lemieux—van Rudloff reagent remained yellow and were not very stable in water, probably due to the presence of manganate ions that had not been completely removed. Therefore, to avoid the possible presence of remaining oxidant in water, we introduced 10 mM ascorbic acid, to reduce the contaminant in one of the purification steps. When transfer to water was performed with RuCl_3_/NaIO_4_ as a catalyst, the final product after complete oxidation was transparent and did not sediment. NaYF_4_:10%Eu^+3^-NPs were well dispersed and they did not aggregate. Therefore, all the following experiments were carried out with NaYF_4_:10%Eu^+3^-NPs oxidized with RuCl_3_/NaIO_4_ reagent.

**Fig 1:**
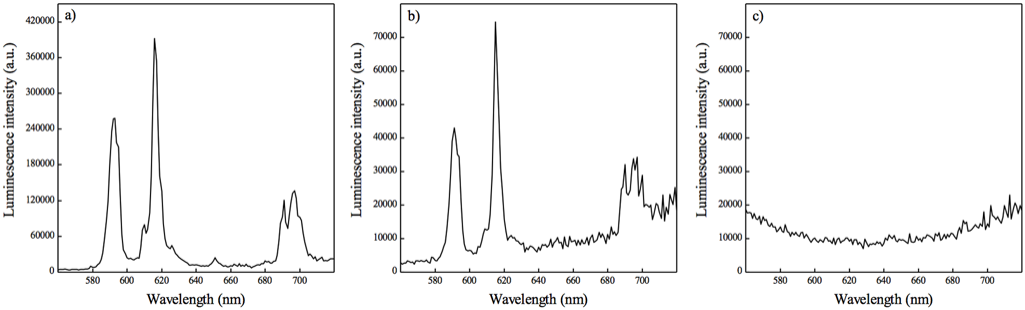
Luminescent properties of NaYF_4_:10%Eu^+3^ NPs. a) emission spectrum of synthesized NaYF4:10%Eu^+3^ NPs in cyclohexane b) emission spectrum of NaYF_4_:10%Eu^+3^ NPs after transfer to water—aqueous fraction c) emission spectrum of the organic fraction remaining after NPs transferred to water. All emission spectra were carried out with the excitation wavelength of 394 nm at room temperature.

To control the efficiency of the oxidation reaction, luminescence measurements were performed. Fig. 1 presents the comparison of Eu^3+^ characteristic emission before and after the transfer of NaYF_4_:10%Eu^+3^-NPs in water. NaYF_4_:10% Eu^+3^-NPs in cyclohexane showed intense peaks at 592, 615, 651 and 696 nm when excited at 394 nm (Fig. 1a). Those bands could be attributed to the consecutive ^5^D_0_→^7^F_J = 1,2,3,4_ f-f electronic transitions in Eu^3+^ ions [11,34]. It is of interest that after the oxidation reaction two separate fractions were distinguished—the organic and the aqueous phases. To control if all the NaYF_4_:10%Eu^+3^-NPs were transferred to water during the reaction, emission spectra of both fractions were measured. Fig. 1b presents the emission spectrum of the aqueous fraction excited at 394 nm. We observed again four characteristic emission bands of Eu^3+^ ions, as was observed in the case of synthesized hydrophobic NaYF_4_:10%Eu^+3^-NPs (Fig. 1a). Moreover, measurement of the emission spectra of the organic fraction after 394 nm excitation (Fig. 1c) did not show any luminescence signals from NaYF_4_:10%Eu^+3^-NPs, indicating that NPs were efficiently transferred into the aqueous fraction. As this fraction did not contain hydrophobic elements, oxidation of oleic acid ligands present on the NaYF_4_:10%Eu^+3^-NPs surface was therefore completed. However, the intensity of the peaks before and after oxidation (Fig. 1a and 1b) decreased most probably due to the loss of material during the purification steps.

### Bio-conjugation

The carboxylic groups created on the surface of NaYF_4_:10%Eu^+3^-NPs upon transfer into water were activated by EDC/NHS to facilitate the covalent binding of 5’ amine-tagged ssDNA [35]. To confirm the formation of amide bonds at the interface, we used DNA radiolabeled at its 3’ extremity. An image and description of agarose gel electrophoresis of NaYF_4_:10%Eu^+3^-NP and DNA complexes can be found in Supporting Information (S3 Fig.).

NaYF_4_:10%Eu^+3^ NPs conjugated with ssDNA-NH_2_ were incubated in PBS buffer with the complementary DNA strand labeled with the fluorescent Cy5 dye at the 5’ end. The length of the complementary DNA strand determined the distance separating the NaYF_4_:10%Eu^+3^-NP surface from the Cy5 fluorophore. Assuming that after hybridization the double strand was a B-DNA tertiary structure, the distance between two base pairs (bp) should correspond to 0.34 nm. Furthermore, the ssDNA-NH_2_ used in all experiments contained additional 5 thymine bases that served as a spacer and an ‘aminolinker’ with six-carbon chain of a total length of around 1 nm. Therefore using oligonucleotides of 26, 31, 41 and 50 base pairs, we obtained a distance range between 11 nm for 26 bp DNA and nearly 20 nm for 50 bp DNA. The estimated distance between the donor and the acceptor, imposed by the length of the complementary DNA strand was further used for comparison with values obtained based on spectroscopic measurements and FRET formalism [27].

### Energy transfer efficiency measurements

**Fig 2:**
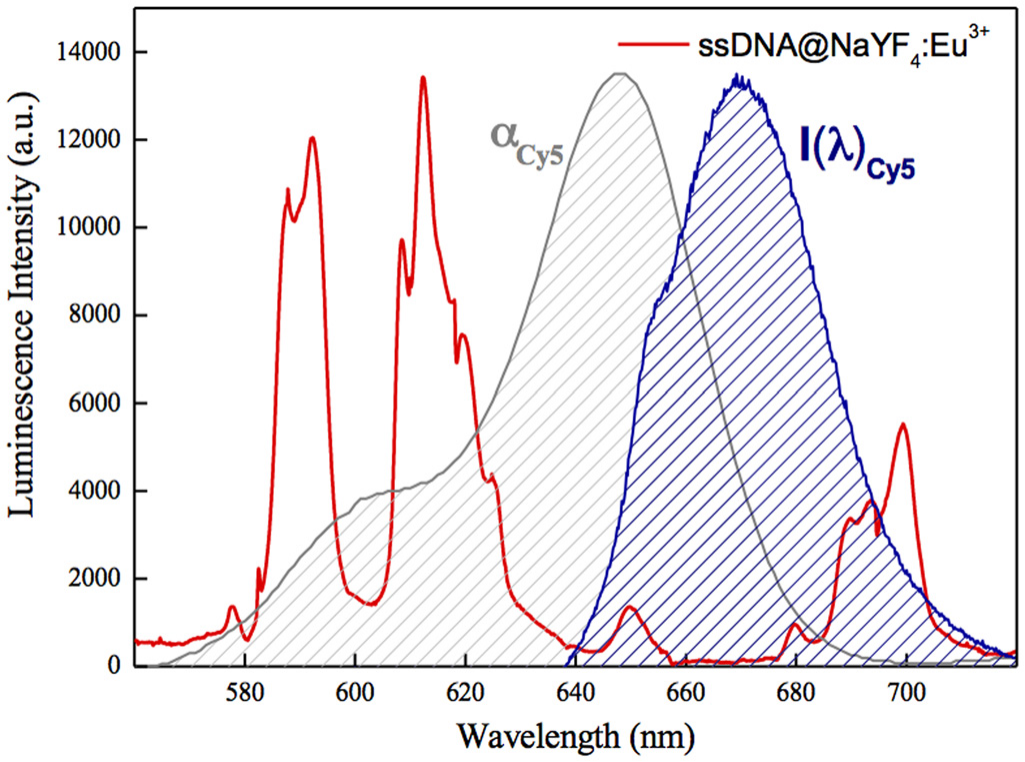
Luminescence spectra of NaYF_4_:10%Eu^3+^ NPs-ssDNA-NH_2_ complex excited with 394 nm laser light (red curve). The absorption (α_Cy5_, grey shaded curve) and emission (I(λ)_Cy5_, blue shaded curve) spectra under 650 nm excitation for Cy5 fluorophore are presented for comparison.

Cy5 as a DNA end labelling dye was chosen because its absorption band overlaps two main emission bands of NaYF_4_:10%Eu^3+^-NPs (^5^D_0_→^7^F_2_ and ^5^D_0_→^7^F_1_) (Fig. 2). The laser light used for this experiment (λ = 394 nm) to excite the Eu^3+^ ions luminescence did not cause absorption from Cy5 molecules. Based on the spectroscopic features of separate components of the systems studied, conditions should be sufficient for NaYF_4_:10%Eu^3+^-NPs → Cy5 dye FRET or energy reabsorption to occur. In order to draw conclusions about the dominating mechanism we performed a detailed spectroscopic investigation of the prepared hybrid systems.

**Fig 3:**
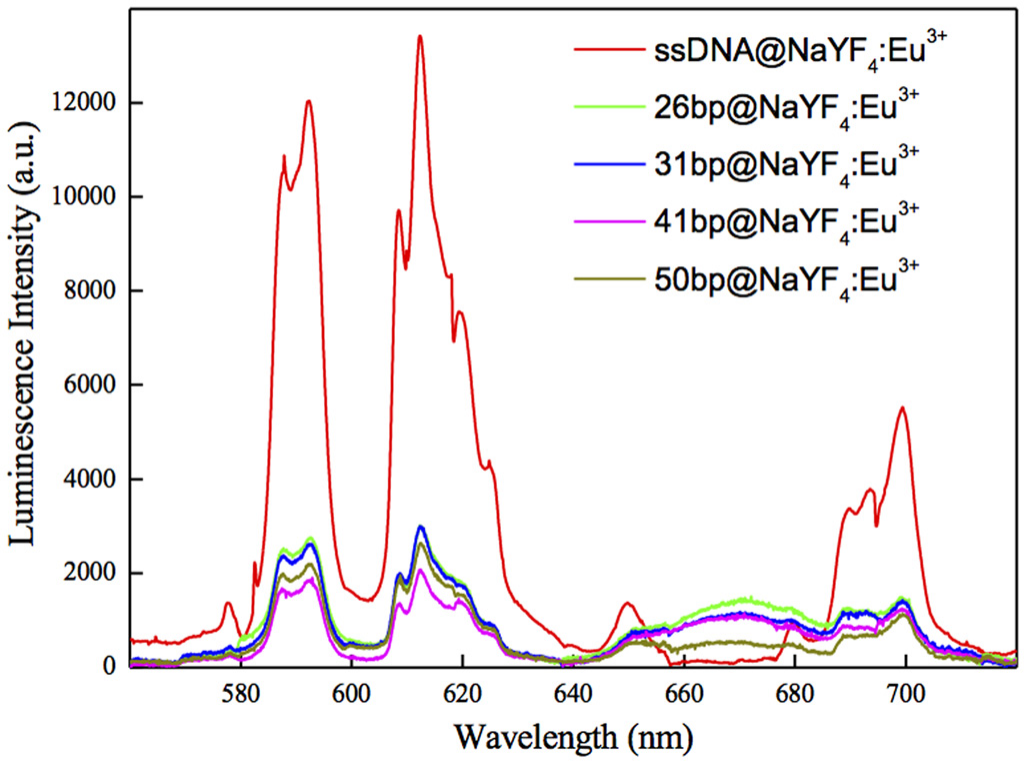
Luminescence spectra of NaYF_4_:10%Eu^3+^ NP-DNA hybrid systems. The ssDNA@NaYF_4_:Eu^3+^ sample (red curve) is considered as a reference. The 26bp@NaYF_4_:Eu^3+^ (green), 31bp@NaYF_4_:Eu^3+^ (blue), 41bp@NaYF_4_:Eu^3+^ (magenta) and 50bp@NaYF_4_:Eu^3+^ (yellow) samples correspond respectively to luminescence spectra of the complexes NaYF_4_:10%Eu^3+^NP- with 26bp, 31bp, 41bp and 50 bp dsDNA.

First, we measured the luminescence spectra of hybrid systems having Cy5 dye molecules at different fixed distances from NPs surfaces. It must be borne in mind, that we could not fully exclude DNA bending and such a situation most probably occurred in the systems under study, and may have introduced some degree of inaccuracy in estimating distances between NaYF_4_:10%Eu^+3^-NPs and Cy5 molecules. Additionally, we assumed an equal concentration of the NaYF_4_:10%Eu^+3^-NPs in samples containing dsDNA-Cy5. The results of luminescence spectra measurements are presented in Fig. 3.

**Fig 4:**
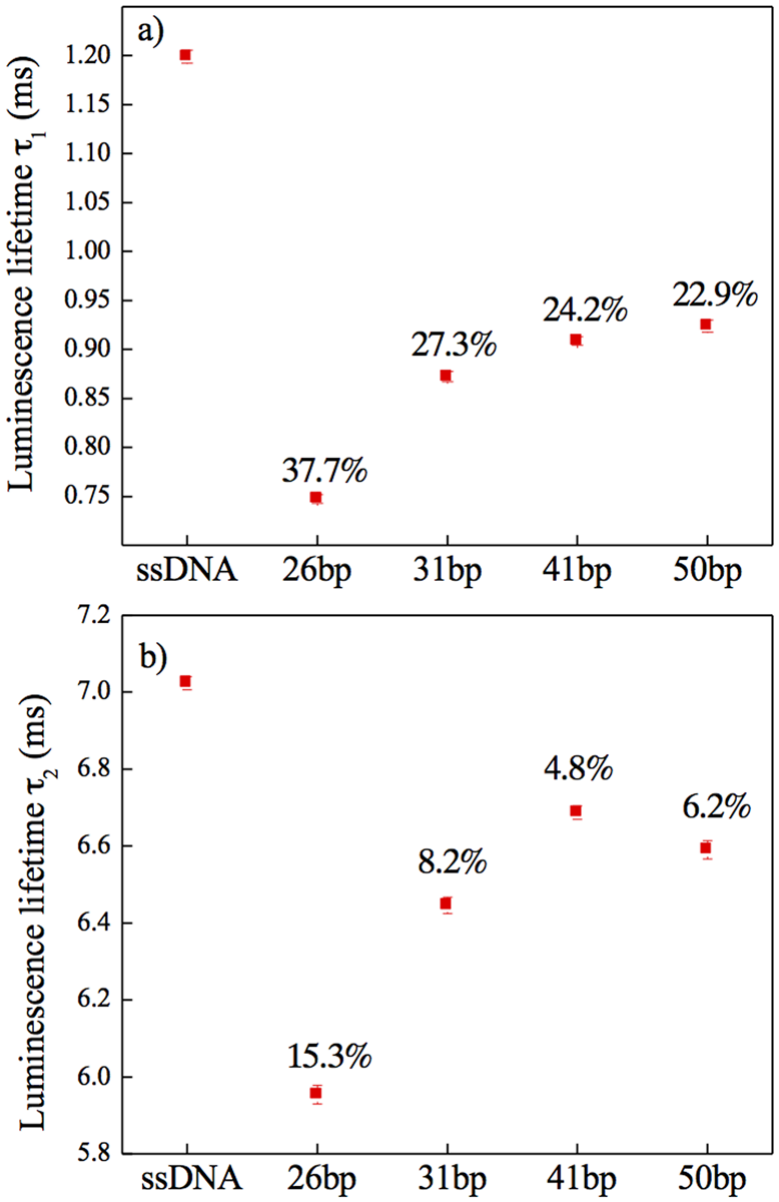
Luminescence lifetime. a) Long and b) short components of luminescence lifetime values measured at 612 nm for the ^5^D_0_→^7^F_2_ emission band in Eu^3+^ ions in hybrid systems consisting of 10% Eu^3+^ doped NaYF_4_ NPs and DNA molecules bound to the NPs surface. The wavelength selection was performed with a JobinYvon THR1000 monochromator. Included numbers are the energy transfer efficiencies, η calculated based on the FRET mechanism.

Hybridization of complementary DNA strands tagged with Cy5 dye resulted in an overall decrease of Eu^3+^ luminescence and in the appearance of an additional band in the emission spectra. This broad band in the range of 640–680 nm was attributed to Cy5 emission. The increasing distance between NaYF_4_:10%Eu^+3^-NPs and Cy5 fluorophore molecules resulted in a decrease of Cy5 luminescence. In other words, the observed decreased intensity of NaYF_4_:10%Eu^3+^-NPs emission together with the appearance of the Cy5 emission band in the spectra should be due to energy transfer between the donor and the acceptor. However, measurements of only luminescence spectra are inconclusive concerning the dominant mechanism present in the system. Additionally, some degree of concentration fluctuation of optically active species in investigated samples, as well as fluctuation of the excitation intensity needs to be taken into account. These factors can greatly influence the results of luminescence spectra measurements, and thus make the experimental data insufficient to clearly confirm FRET based energy transfer. The luminescence lifetime values of NaYF_4_:10%Eu^3+^-NPs with bound ssDNA-NH_2_, as well as those for hybridized complementary fluorescent-tagged DNA strands of different length should provide more detailed information concerning the dominant mechanism. The measured luminescence decays were double exponential. The presence of Cy5 molecules resulted in a decrease of both long and short components (Fig. 4a and 4b respectively) of the ^5^D_0_ excited state lifetime of Eu^3+^ doped NaYF_4_-NPs. The representative decay curves for ssDNA@NaYF_4_:10%Eu3+-NPs, 26bp@NaYF4:10%Eu3+-NPs, 31bp@NaYF4:10%Eu3+-NPs, 41bp@NaYF4:10%Eu3+-NPs and 50bp@NaYF4:10%Eu3+-NPs are [Apex: for all instances “YF4” subscript 4; for “Eu3+” superscript 3+] presented in Supporting Information (S4 Fig.). This can be taken as direct evidence of nonradiative energy transfer. Additionally, the shortening of the lifetime was most effective for the shortest dsDNA (26 bp), in which the Cy5 fluorophore was closest to the surface of Eu^3+^ doped NPs. Increasing the Eu^3+^ doped NP→Cy5 distance resulted in a smaller luminescence lifetime change. Based on FRET formalism and luminescence lifetime measurements, we were able to calculate the energy transfer efficiencies:

η=1-τDAτD

where τ_*D*_ is the lifetime of the donor in the presence of the acceptor, and τ_*D*_ is the lifetime of the donor in the absence of the acceptor molecules.[36] As can be seen from data shown in Fig. 4 the highest energy transfer efficiency was observed for the shortest dsDNA-Cy5 (26 bp).

In order to estimate the distance range over which energy transfer was most efficient we calculated the Förster radius (R_o_ [Å]), at which η = 50%. Details concerning the calculation can be found in S2 Supporting Information. Taking extreme values of the R_0_ (S5 Fig.) and the measured energy transfer efficiencies (η) for short lifetime components, we calculated theoretical distances between NaYF_4_:10%Eu^3+^ and Cy5 dye molecules (r) [36].

$$\eta =1-\frac{\tau_{DA}}{\tau_{D}}=\frac{R_{0}^{6}}{R_{0}^{6}+r^{6}}$$

These fell in the ranges: 8.4 nm < r_26bp_ > 16.3 nm, 10.6 nm < r_31bp_ > 17.7 nm, 10.9 nm < r_41bp_ > 18.1 nm and 11.0 nm < r_50bp_ > 18.4 nm. The results of calculations of r values could be compared with the estimated real nano-scale distances from NaYF_4_:10%Eu^+3^-NPs surfaces to Cy5 dye. Assuming that donor and acceptor were separated with azelaic acid, ‘amino linker’, 5 thymines and dsDNA of lengths 26 bp, 31 bp, 41 bp and 50 bp, the distances were ~11 nm, ~13 nm, ~17 nm and ~20 nm, respectively, which is in good agreement with the values obtained from FRET based calculations. The experimental results converge with the performed calculations confirming a nonradiative energy transfer mechanism in these systems.

Finally, we suggest that ultra-small Eu^3+^ doped NaYF_4_-NPs may be useful as nano-rulers for measuring distances on a nanometric scale.

### Controlled decrease of FRET—enzymatic digestion of DNA

**Fig 5:**
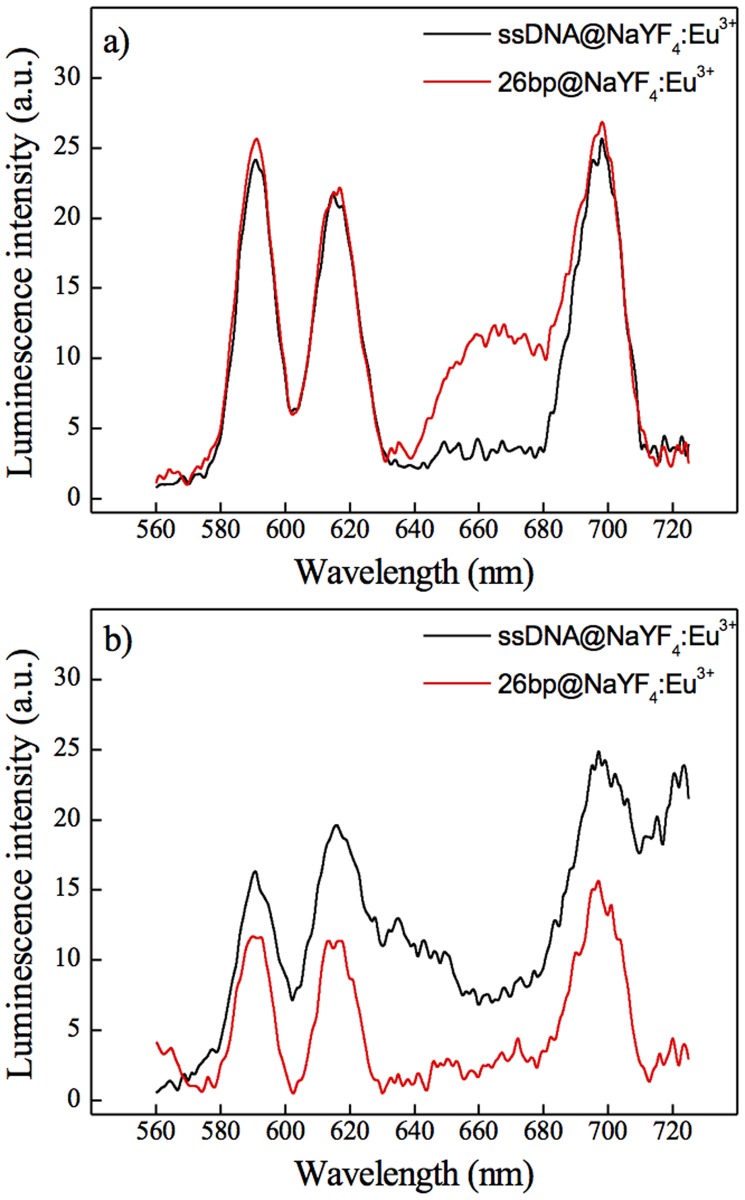
Emission spectra of the 26bp hybrid system upon enzymatic digestion. **a)** before digestion, **b)** after enzymatic digestion with BamHI. NaYF_4_:10%Eu^3+^ NPs conjugated with ssDNA-NH_2_ and the complementary (red curve) and non-complementary (black curve) DNA strand with Cy5 tag. Samples were excited at the wavelength of 394 nm.

The presence of an additional band at around 670 nm (Fig. 3) and lifetime measurements (Fig. 4) confirmed that nonradiative energy transfer occurred. However, in order to demonstrate both the specificity of DNA binding to the surface, and the accessibility/integrity of bound DNA, complexes were incubated with BamHI restriction enzyme. As a result, the dsDNA was cut and the fluorescent fragment released into the solution. A complex of NaYF_4_:10%Eu^+3^-NPs-ssDNA-NH_2_ incubated with a non-complementary ssDNA-Cy5 (sequences are listed in S1 Table) was taken as a control. Comparison of emission spectra before and after digestion (Fig. 5) indicated that the wide band (at around 670 nm) disappeared after digestion. Complete loss of this peak after digestion of dsDNA-Cy5 by BamHI and lack of the signal at 670 nm in the case of the control sample confirmed that dsDNA attached to the surface was accessible for further biological reactions.

## Conclusions

We have demonstrated an efficient method to transfer ultra-small Eu^3+^ doped NaYF_4_ NPs dispersed in organic solvent to an aqueous environment via oxidation of the oleic acid ligand by using Lemieux—van Rudloff reagent and its analogue. We present an easy and efficient means of functionalizing NaYF_4_:10%Eu^3+^-NPs with DNA by inducing covalent bonds between COOH groups on the La-NPs surface and NH_2_ tagged DNA. Our results demonstrate that nonradiative energy transfer occurs between Eu^3+^ ions doped in the crystal nanostructure of NaYF_4_-NPs and Cy5 fluorophores placed at the end of dsDNA strands. Different lengths of DNA were chosen in order to show the dependence of energy transfer efficiency on the distance between the donor and the acceptor in a range from 11 to 20 nm. A new approach based on results presented in this paper clearly indicates that efficient FRET using Eu^3+^ ions as donor energy is a robust technique giving accurate and reproducible results [28–31]. Enzymatic digestion of restriction sites of NP bound dsDNA additionally confirmed the functionality of probes and the stability of the complexes. This study provides a foundation for the development of biodetecting systems for advanced biophotonics applications.

## Supporting Information

S1 Fig Transmission Electron Microscopy (TEM) image of NaYF_4_:10%Eu^+3^ NPs. a) in chloroform, b) after transfer to water.
(TIFF)

S2 Fig Size distribution of NaYF_4_:10%Eu^+3^ NPs. a) in chloroform, b) after transfer to water. Each of the results presented represents an average of 6 repeated measurements.
(TIFF)

S3 Fig 1% agarose electrophoresis gel of complexes of NaYF_4_:10%Eu^+3^ NPs and radioactive labeled DNA. 1- NPs conjugated with ssDNA-NH_2_, 2- NPs conjugated with ssDNA-NH_2_ + 50bp-Cy5 Complementary ssDNA, 3- NPs conjugated with ssDNA-NH_2_ + 50bp-Cy5 non-complementary ssDNA.
(TIFF)

S4 Fig Luminescence decay curves of NaYF_4_:10%Eu^+3^ NPs conjugated. With ssDNA-NH_2_ (a) and complexes of NaYF_4_:10%Eu^+3^ NPs and Cy5 fluorescent tag placed at the end of 26 bp dsDNA (b), 31 bp dsDNA (c), 40 bp dsDNA (d) and 51 bp dsDNA (e). The decay curves were measured at 612 nm for the ^5^D_0_→^7^F_2_ emission band in Eu^3+^ ions, with the wavelength selection was performed by a JobinYvon THR1000 monochromator.
(TIFF)

S5 Fig Förster distances as a function of NaYF_4_:10%Eu^3+^ NPs quantum yield. **(*QY*)**, calculated for different relative molecular orientation of donor and acceptor: *κ* ^2^.
(TIFF)

S1 Supporting Information (DOCX)

S2 Supporting Information (DOCX)

S1 Table Sequences of oligonucleotides Consensus recognition sites for BamHI are underlined. Sequences numbered 2-5 and 6 correspond respectively to complementary and non-complementary single stranded DNA with respect to the ssDNA attached to the NaYF_4_:10%Eu^+3^-NPs surface.
(DOCX)
