# Supplementary figures and images for: DNA Base Pair Resolution Measurements Using Resonance Energy Transfer Efficiency in Lanthanide Doped Nanoparticles
Source: PLoS One. 2015 Mar 6;10(3):e0117277. doi: 10.1371/journal.pone.0117277 (PMC4351948; doi:10.1371/journal.pone.0117277)

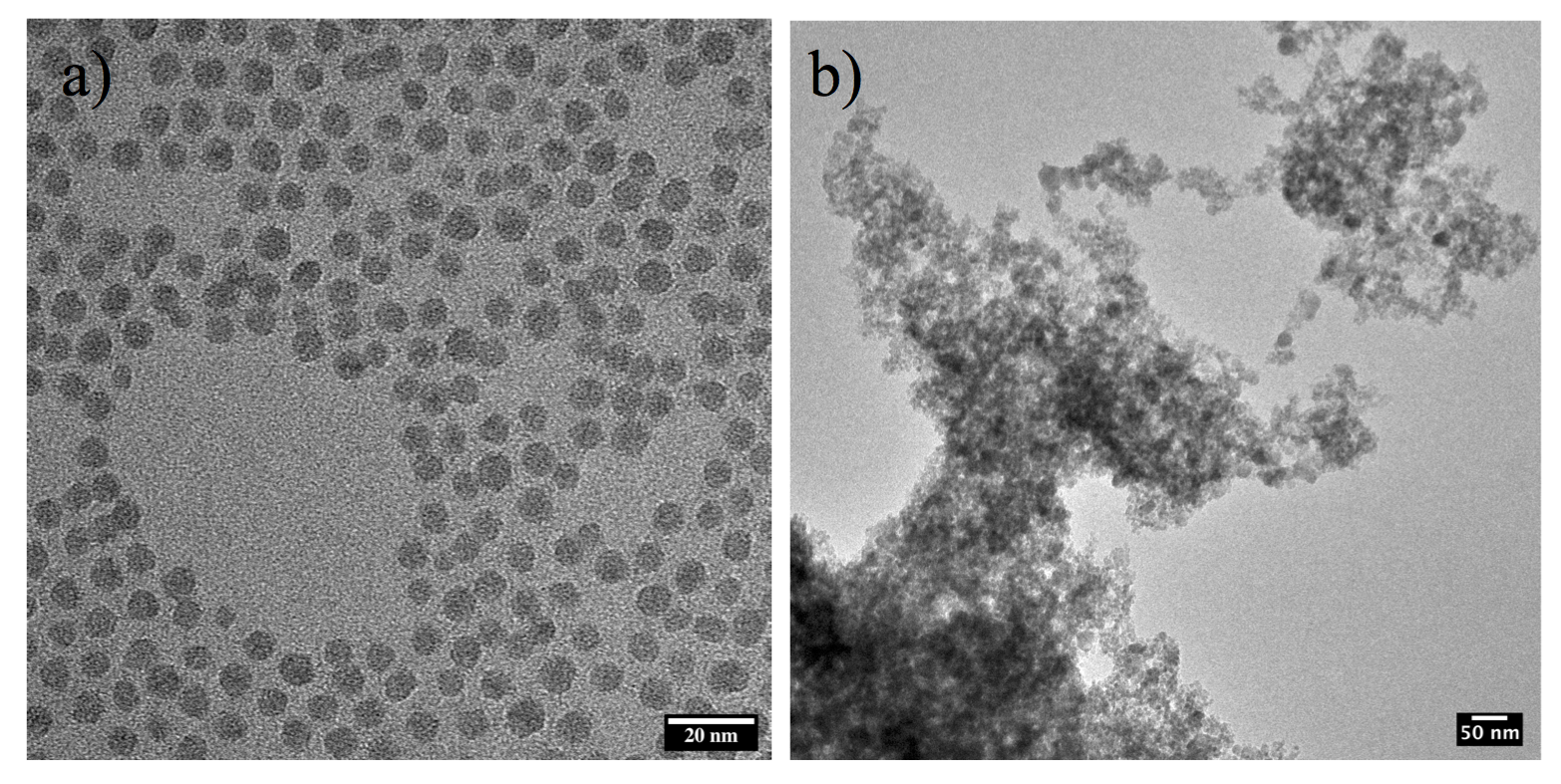

Supplement: S1 Fig — a) in chloroform, b) after transfer to water. (TIFF) [file pone.0117277.s001.tiff]

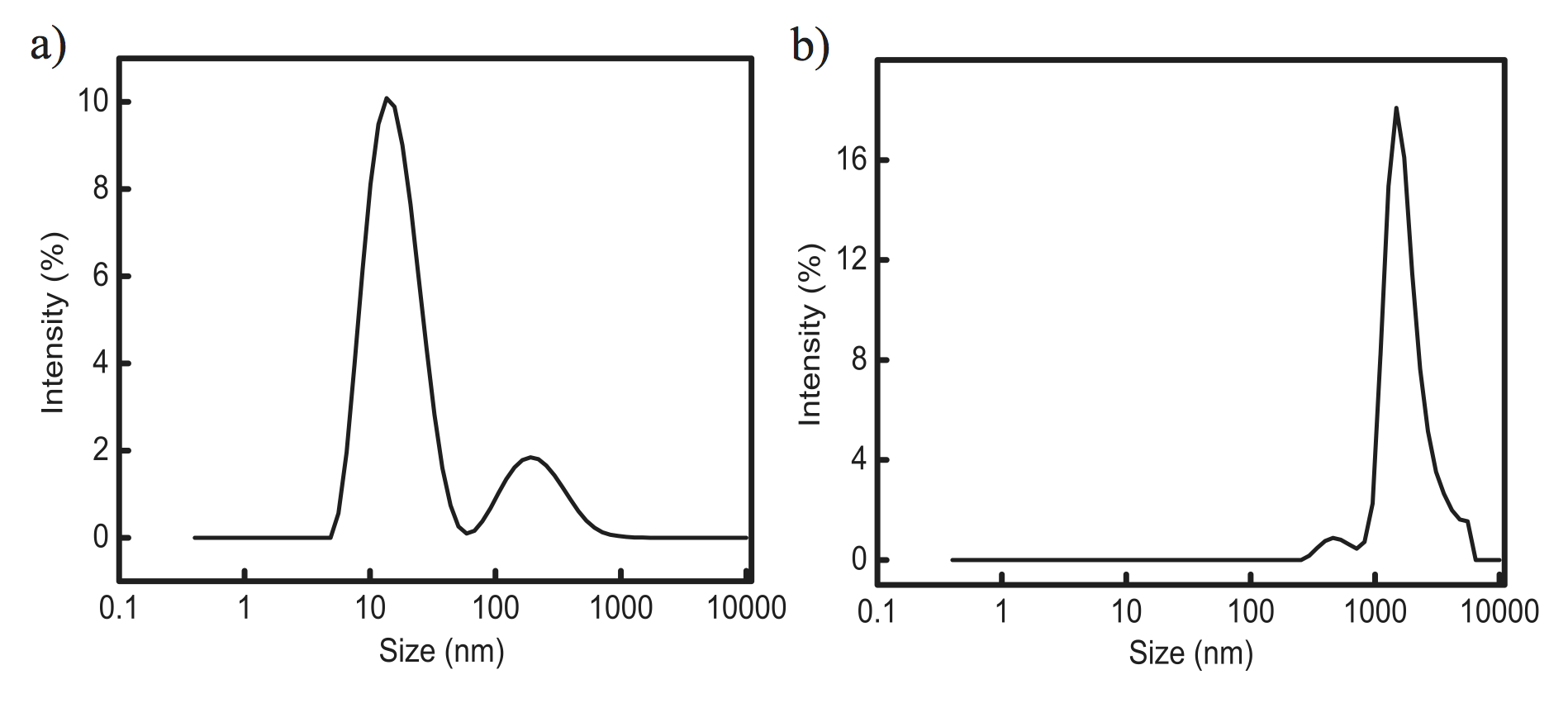

Supplement: S2 Fig — a) in chloroform, b) after transfer to water. Each of the results presented represents an average of 6 repeated measurements. (TIFF) [file pone.0117277.s002.tiff]

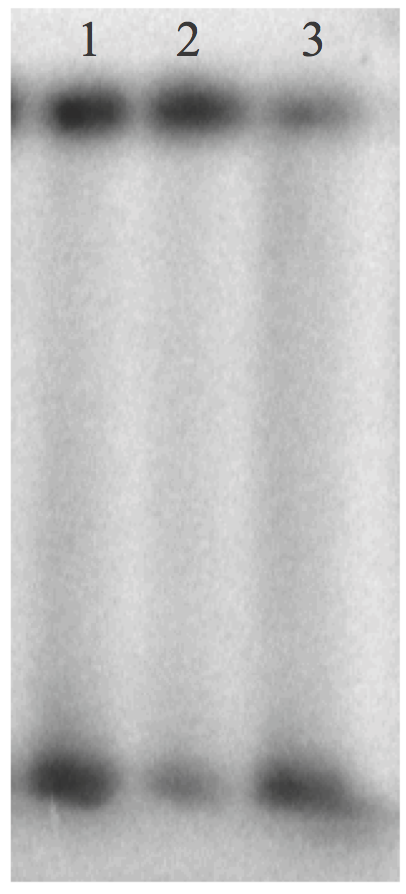

Supplement: S3 Fig — 1- NPs conjugated with ssDNA-NH2, 2- NPs conjugated with ssDNA-NH2 + 50bp-Cy5 Complementary ssDNA, 3- NPs conjugated with ssDNA-NH2 + 50bp-Cy5 non-complementary ssDNA. (TIFF) [file pone.0117277.s003.tiff]

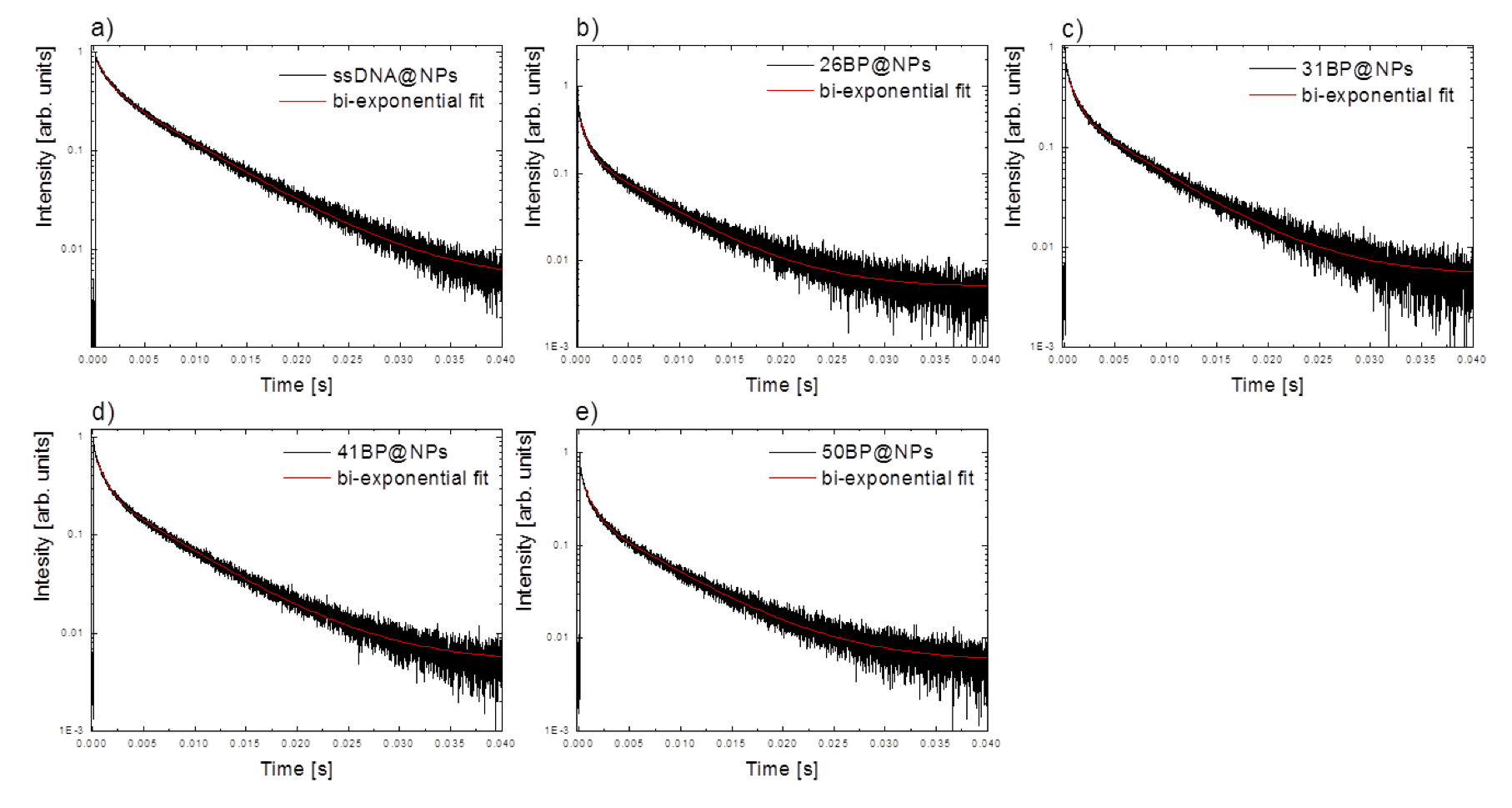

Supplement: S4 Fig — With ssDNA-NH2 (a) and complexes of NaYF4:10%Eu+3 NPs and Cy5 fluorescent tag placed at the end of 26 bp dsDNA (b), 31 bp dsDNA (c), 40 bp dsDNA (d) and 51 bp dsDNA (e). The decay curves were measured at 612 nm for the 5D0→7F2 emission band in Eu3+ ions, with the wavelength selection was performed by a JobinYvon THR1000 monochromator. (TIFF) [file pone.0117277.s004.tiff]

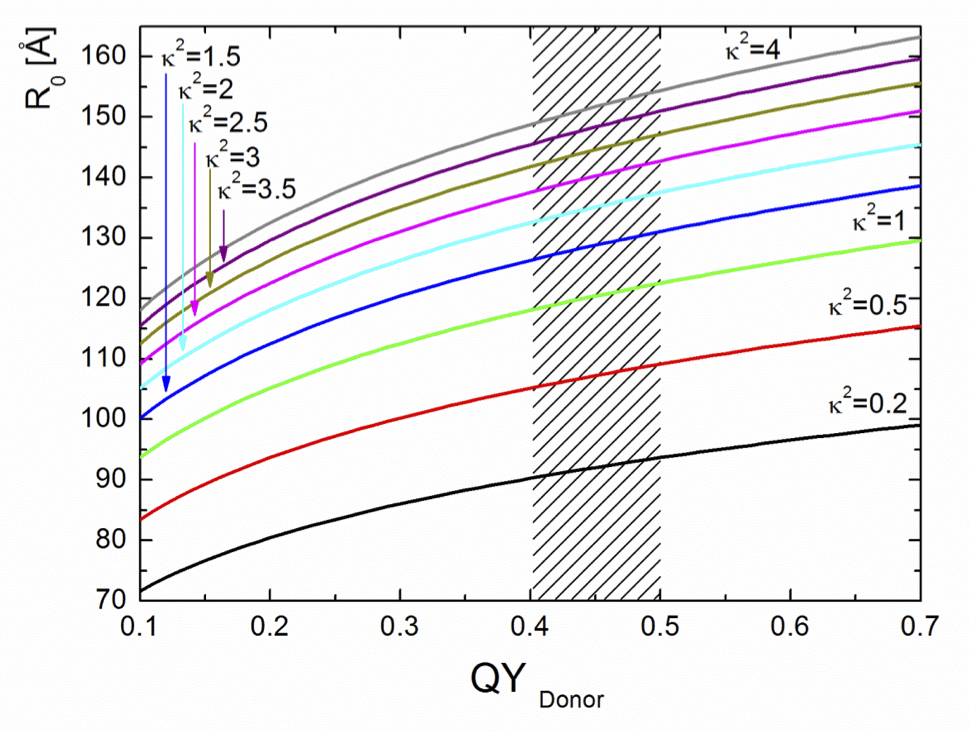

Supplement: S5 Fig — (QY), calculated for different relative molecular orientation of donor and acceptor: κ 2. (TIFF) [file pone.0117277.s005.tiff]
