# Supplementary material for: DNA Base Pair Resolution Measurements Using Resonance Energy Transfer Efficiency in Lanthanide Doped Nanoparticles
Source: PLoS One. 2015 Mar 6;10(3):e0117277. doi: 10.1371/journal.pone.0117277 (PMC4351948; doi:10.1371/journal.pone.0117277)
Supplement: S1 Supporting Information — (DOCX) [file pone.0117277.s006.docx]

**Supporting Information, Section S1**

**Bio functionalization:** The surface of NaYF_4_:10%Eu^+3^ NPs after transfer into water was functionalized in order to covalently bind DNA molecules. EDC/NHS was used to facilitate binding DNA with an amine tag. To confirm the presence of amide bonds, radiolabelled DNA was used. Labeling of ssDNA-NH_2_ allowed bond formation between COOH groups on the NP surface and amine-tagged DNA. To confirm the accessibility of ssDNA bound to the surface, we hybridized a complementary strand of radiolabeled DNA. Noncomplementary ssDNA (sequence as in ‘noncomp_Cy5’ oligonucleotide, Table S1) was used as a control. All samples were run in a 1% agarose gel electrophoresis (Fig. S3). The electrophoresis image confirmed the presence of two bands. The upper bands represent DNA bound to NPs. The majority of NP-DNA complexes did not enter into the 1% agarose gel presumably due either to the large size of the complexes or/and by an unfavorable global charge. The lower bands represent the excess of DNA not bound to the surface but not removed during the sample rinsing (samples were rinsed 3 times). Smears in the electrophoresic pattern were caused by different stoichiometries of DNA molecules bound to certain NPs.
