# Supplementary material for: DNA Base Pair Resolution Measurements Using Resonance Energy Transfer Efficiency in Lanthanide Doped Nanoparticles
Source: PLoS One. 2015 Mar 6;10(3):e0117277. doi: 10.1371/journal.pone.0117277 (PMC4351948; doi:10.1371/journal.pone.0117277)
Supplement: S2 Supporting Information — (DOCX) [file pone.0117277.s007.docx]

**Supporting Information, Section S2**

Förster radius ($R_{0}$) depends on: the quantum yield of the donor in the absence of the acceptor ($QY$), the overlap integral ($j$) of the donor emission spectrum and the acceptor absorption spectrum, their relative molecular orientation ($\kappa^{2}$) and the refractive index of the medium ($n$) as expressed by the following equation:

$$R_{0}^{6}=8,79\times10^{-5}\times n^{-4}\times QY\times\kappa^{2}\times j$$

where the spectral overlap integral: $j=\int_{0}^{\infty} f_{D}\left(\lambda\right)\varepsilon_{A}\left(\lambda\right)^{4}d\lambda$ includes normalized donor emission$f_{D}\left(\lambda\right)$ and molecular absorption coefficient spectra of the acceptor $\varepsilon_{A}\left(\lambda\right)$. For the hexagonal phase, 5% Eu^3+^ doped ultra-small NaYF_4_ NPs the $QY$ was reported to be around 55% [[1](#_ENREF_1)]. On the other hand, Kim *et al*. [[2](#_ENREF_2)] reported much lower $QY=1.7\%$ values for 5 nm sized β-Na(Y,Gd)F_4_:10%Ce^3+^,15%Tb^3+^,10%Eu^3+^ NPs. In general, the $QY$ value depends on the type of host material, NPs size, crystal structure, surface coatings, the amount and the type of the active ions embedded in the matrix, and it should be estimated separately for a particular examined material. In our experiments we used 10% Eu^3+^ doped samples with ultra-small size below 10 nm, so the $QY$ is most probably lower than reported by Banski *et al*. [[1](#_ENREF_1)] due to quenching by the increased concentration. On the other hand, we used direct Eu^3+^ excitation, which should increase the $QY$ value in comparison to the value reported for co-doped system where the sequence energy transfer between Ce^3+^ ions to Tb^3+^ or Gd^3+^ ions to the Eu^3+^ ions occurs [[2](#_ENREF_2)]. Another fact, which should be taken into consideration when calculating the $R_{0}$ values, is the relative orientation of donor and acceptor molecules. For most cases $\kappa^{2}$ is assumed to be 2⁄3, which indicates that donor and acceptor molecules are freely rotating and can be considered to be isotropically oriented during the excited state lifetime [[3](#_ENREF_3)]. In our case the acceptor molecules are attached to the donor NPs surface through DNA strands, and most probably do not reorient in the Eu^3+^ luminescence lifetime time scale. Such an assumption would require setting the $\kappa^{2}$ values to a broader range: 0≤ $\kappa^{2}$ ≥4. Considering what was discussed above, we performed the calculation of the $R_{0}$ values for different $QY$ and $\kappa^{2}$ values, and present the results in Figure S5. Assuming the $QY$ values in the range 0.4 to 0.5 results in 9 nm < $R_{0}$ > 15 nm.

**References:**

1. Banski M, Afzaal M, Podhorodecki A, Misiewicz J, Abdelhady AL, et al. (2012) Passivation of lanthanide surface sites in sub-10 nm NaYF4:Eu3+ nanocrystals. Journal of Nanoparticle Research 14.

2. Kim SY, Woo K, Lim K, Lee K, Jang HS (2013) Highly bright multicolor tunable ultrasmall beta-Na(Y,Gd)F-4:Ce,Tb,Eu/beta-NaYF4 core/shell nanocrystals. Nanoscale 5: 9255-9263.

3. Bednarkiewicz A, Nyk M, Samoc M, Strek W (2010) Up-conversion FRET from Er3+/Yb3+:NaYF4 Nanophosphor to CdSe Quantum Dots. Journal of Physical Chemistry C 114: 17535-17541.
