# Supplementary material for: DNA Base Pair Resolution Measurements Using Resonance Energy Transfer Efficiency in Lanthanide Doped Nanoparticles
Source: PLoS One. 2015 Mar 6;10(3):e0117277. doi: 10.1371/journal.pone.0117277 (PMC4351948; doi:10.1371/journal.pone.0117277)
Supplement: S1 Table — Consensus recognition sites for BamHI are underlined. Sequences numbered 2-5 and 6 correspond respectively to complementary and non-complementary single stranded DNA with respect to the ssDNA attached to the NaYF4:10%Eu+3-NPs surface. (DOCX) [file pone.0117277.s008.docx]

**Supplementary Information, S1 Table**

**Sequences of oligonucleotides.**

| Name | | 5’ modification | Sequence (5’ to 3’) |
| --- | --- | --- | --- |
| 1 | ssDNA-NH_2_  ssDNA-OH | NH_2_(C6)/OH | TTT TTT CCA TCA GTC ACT CGC CGG ATC CAT CTT GAG ACT CTG TCA CAC GTA GTC G |
| 2 | 26bp-Cy5 | Cy5 | GAT GGA TCC GGC GAG TGA CTG ATG GA |
| 3 | 31bp-Cy5 | Cy5 | CTC AAG ATG GAT CCG GCG AGT GAC TGA TGG A |
| 4 | 41bp-Cy5 | Cy5 | GTG ACA GAG TCT CAA GAT GGA TCC GGC GAG TGA CTG ATG GA |
| 5 | 50bp-Cy5 | Cy5 | CGA CTA CGT GTG ACA GAG TCT CAA GAT GGA TCC GGC GAG TGA CTG ATG GA |
| 6 | noncomp-Cy5 | Cy5 | AAG AAA TTT GGA TTA TTG CGC TAT CTG AAG TTT TAG TAC GG |

Consensus recognition sites for BamHI are underlined. Sequences numbered 2-5 and 6 correspond respectively to complementary and non-complementary single stranded DNA with respect to the ssDNA attached to the NaYF_4_:10%Eu^+3^-NPs surface.
